# Signal Recognition Particle and SecA Cooperate during Export of Secretory Proteins with Highly Hydrophobic Signal Sequences

**DOI:** 10.1371/journal.pone.0092994

**Published:** 2014-04-09

**Authors:** Yufan Zhou, Takuya Ueda, Matthias Müller

**Affiliations:** 1 Institute of Biochemistry and Molecular Biology, Zentrum für Biochemie und Molekulare Zellforschung, University of Freiburg, Freiburg, Germany; 2 Faculty of Biology, University of Freiburg, Freiburg, Germany; 3 Department of Medical Genome Sciences, Graduate School of Frontier Sciences, The University of Tokyo, Chiba, Japan; Centre National de la Recherche Scientifique, Aix-Marseille Université, France

## Abstract

The Sec translocon of bacterial plasma membranes mediates the linear translocation of secretory proteins as well as the lateral integration of membrane proteins. Integration of many membrane proteins occurs co-translationally via the signal recognition particle (SRP)-dependent targeting of ribosome-associated nascent chains to the Sec translocon. In contrast, translocation of classical secretory proteins across the Sec translocon is a post-translational event requiring no SRP but the motor protein SecA. Secretory proteins were, however, reported to utilize SRP in addition to SecA, if the hydrophobicity of their signal sequences exceeds a certain threshold value. Here we have analyzed transport of this subgroup of secretory proteins across the Sec translocon employing an entirely defined *in vitro* system. We thus found SecA to be both necessary and sufficient for translocation of secretory proteins with hydrophobic signal sequences, whereas SRP and its receptor improved translocation efficiency. This SRP-mediated boost of translocation is likely due to the early capture of the hydrophobic signal sequence by SRP as revealed by site-specific photo cross-linking of ribosome nascent chain complexes.

## Introduction

The heterotrimeric SecYEG translocon located in the inner membrane of Gram-negative bacteria is used by two different groups of proteins to be exported from the cytoplasm of these organisms: secretory proteins destined for the periplasmic space or the outer membrane are translocated through the pore of the Sec translocon, whereas inner membrane proteins exit laterally from the Sec translocon into the lipid bilayer. Both classes of proteins carry characteristic targeting signals. In the case of secretory proteins these are classical N-terminal signal sequences consisting of a positively charged N-region, a hydrophobic core, and a polar C-region. These signal sequences are typically cleaved off after transport. Membrane proteins instead are recognized via non-cleaved hydrophobic transmembrane helices called signal anchor sequences. For a recent comprehensive review on the structure and function of the Sec translocon, see ref. [1].

The different targeting signals engage two diverse targeting routes. Signal anchor sequences of membrane proteins, which in general are more hydrophobic than classical signal sequences, recruit the signal recognition particle (SRP) consisting of the Ffh protein and the 4.5S RNA in *E. coli*. Binding of SRP occurs co-translationally at the ribosome. The resulting complexes between SRP and ribosome-nascent chains (RNCs) are thought to dock at the membrane-bound SRP receptor (SR), called FtsY in bacteria, via a direct interaction between the Ffh and FtsY proteins [2], from where RNCs are then handed over to the SecYEG translocon [3]. Alternatively, an SRP-FtsY complex might initially form at the membrane in the absence of substrate and only subsequently bind nascent membrane proteins for delivery to the Sec translocon [4]. Still other models of membrane targeting propose a dominant role of the encoding mRNAs and ribosomes prebound to the membrane [5].

On the contrary, the comparably less hydrophobic signal sequences of secretory proteins are not stably bound by SRP at the ribosome and hence elicit a post-translational targeting mode. This usually involves protection by chaperones such as SecB and Trigger factor until the signal sequence binds to the motor protein SecA that has a high affinity for the Sec translocon.

SecA was, however, found to be required also for the assembly of SRP-dependent inner membrane proteins if these harbor extended hydrophilic loops that need to cross the membrane during assembly [6], [7], [8], [9], [10], [11], [12], [13]. In this case, SecA was shown to interact with SecYEG-targeted nascent chains while they are still ribosome-associated and to push a hydrophilic loop across the translocon [12]. A simultaneous dependence on both, SRP and SecA also seems to apply to otherwise secretory proteins equipped with exceedingly hydrophobic signal sequences because export of these naturally SecA-dependent proteins was found to be impaired upon depletion of SRP [14], [15], [16].

In order to be able to discriminate between SRP- and SecA-dependent functions during targeting and translocation of secretory proteins with highly hydrophobic signal sequences we set up an *in vitro* system from entirely defined components. This system was based on the PURE (Protein synthesis Using Recombinant Elements) translation system [17], [18], [19], [20] supplemented with SecYEG-containing proteoliposomes and purified targeting factors. Our studies reveal that, whilst SecA is sufficient for translocation of secretory proteins with hydrophobic signal sequences, SRP and its receptor FtsY improve translocation efficiency by enabling co-translational membrane targeting.

## Results

### Studying Targeting and Translocation of Secretory Proteins with Hydrophobic Signal Sequences under Defined Conditions

**Figure 1:**
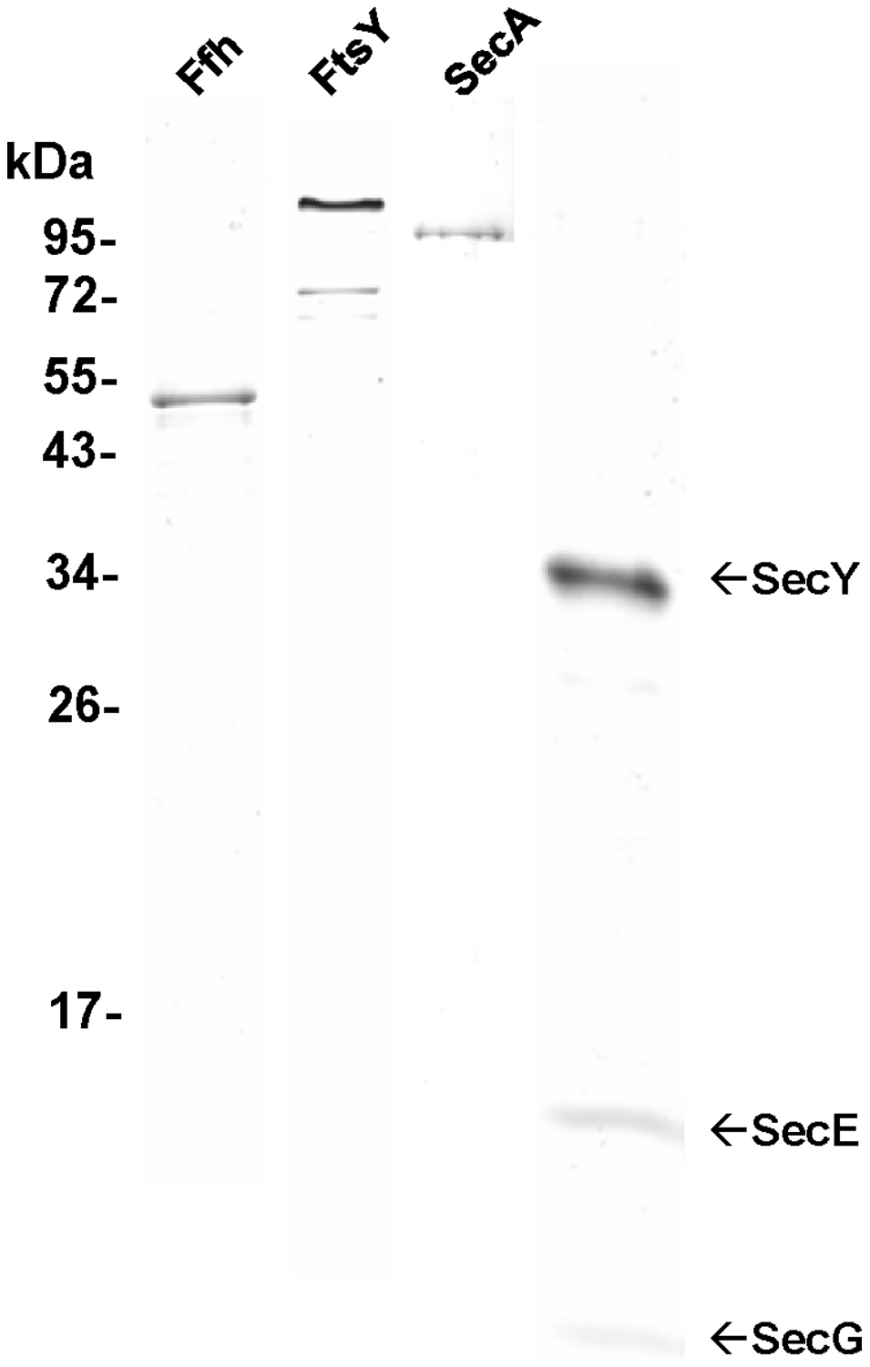
Purification of Ffh, FtsY, SecA and SecYEG complex. His-tagged variants of Ffh, FtsY, and SecA were over-expressed in *E. coli* and purified from cell extracts by metal affinity chromatography. The SecYEG complex was purified by metal affinity chromatography using a DDM-solubilized membrane pellet obtained from a SecY^His^EG-over-producing *E. coli* strain. Purified proteins were displayed by SDS-PAGE and staining with Coomassie Blue.

As examples of secretory proteins harboring pronouncedly hydrophobic signal sequences we chose to study the *E. coli* proteins SfmC and TorT [14] (Table 1). The genes of both proteins were subcloned under the T7 RNA polymerase promoter of vector pET22b(+) and expressed by the PURE system. The PURE system was prepared from the individually purified translation factors and aminoacyl-tRNA synthetases of *E. coli* [18], commercial T7 RNA polymerase, and high salt-washed and membrane-deprived ribosomes. To study translocation of SfmC and TorT across the SecYEG translocon, the SecYEG complex as well as Ffh, FtsY, SecA were all purified to homogeneity from *E. coli* (Figure 1). The DDM-solubilized SecYEG complex was reconstituted with *E. coli* phospholipids into small proteoliposomes by dialysis followed by sonication of the vesicles. The amounts of SecYEG complex reconstituted with a given amount of phospholipids and the volume of SecYEG proteoliposomes to be used per transport assay were each optimized to give maximal translocation efficiency of SfmC.

**Table 1:** Amino acid sequence of signal sequences used.

OmpA	MKKTAIAIAVALAGFATVAQA-
SfmC	MMTKIKLLMLIIFYLIISASAHA-
TorT	MRVLLFLLLSLFMLPAFS-

### SecA is Sufficient to Transport a Secretory Protein with Hydrophobic Signal Sequence across SecYEG, but Efficient Translocation Requires SRP and SR in Addition

**Figure 2:**
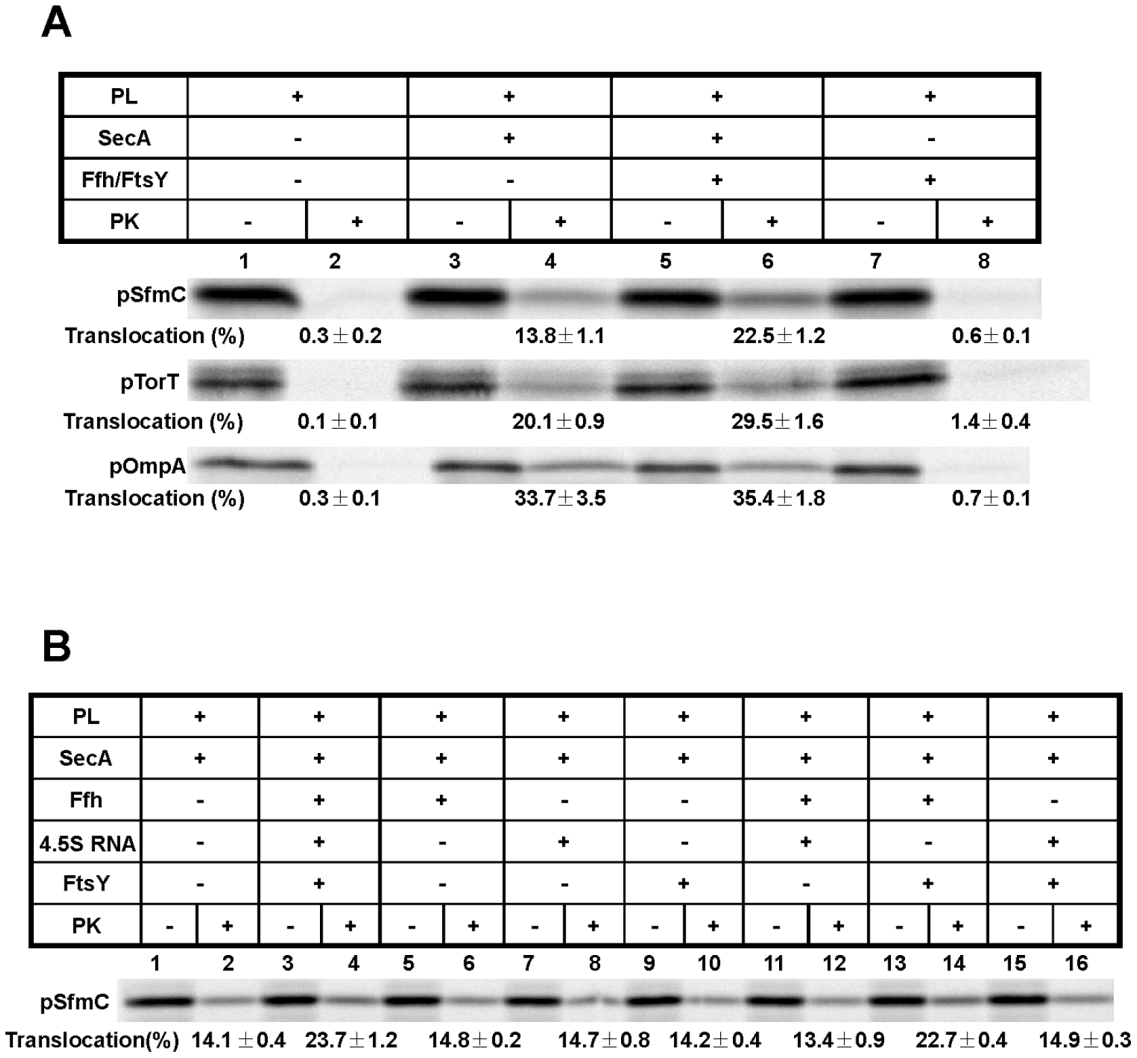
The precursors of SfmC and TorT harbouring hydrophobic signal sequences require SRP and FtsY in addition to SecA for maximal translocation. (**A**) The ^35^[S]-labeled precursors of SfmC (*pSfmC*), TorT (*pTorT*), and OmpA (*pOmpA*) were synthesized by the PURE system in the presence of SecYEG-containing proteoliposomes (*PL*) and purified *SecA, Ffh, FtsY* proteins (1 μg each) as indicated. All components were mixed on ice prior to starting reactions by incubation at 37°C for 1 h. Radiolabeled translation products were separated by SDS-PAGE and are displayed by phosphorimaging. Translocation into the proteoliposomes is indicated by the relative amount of each precursor transformed into a proteinase K (*PK*)-resistant species as determined by measuring the intensities of the corresponding bands using ImageQuant 5.2 (GE-Healthcare). Mean values obtained from three independent experiments and standard errors of the means are given. (**B**) as in (A), except that Ffh, FtsY, and isolated 4.5S RNA were added individually as indicated.

Figure 2A compares translocation of the precursors pSfmC and pTorT into SecYEG proteoliposomes with that of the classical secretory protein OmpA. Translocation was measured by the fraction of each precursor that became resistant towards digestion by proteinase K (PK) in the presence of SecYEG proteoliposomes. In the presence of SecA, about 34% of pOmpA was found translocated into SecYEG proteoliposomes (lane 4). As expected for the SRP/SR-independent OmpA protein, Ffh plus FtsY neither significantly stimulated the SecA-mediated translocation efficiency (lane 6) nor were they able to replace SecA (lane 8). In contrast, maximal translocation efficiencies of both pSfmC and pTorT were only obtained in the presence of Ffh, FtsY and SecA (lane 6). SecA was indispensable for translocation of pSfmC and pTorT (lane 8), but different from pOmpA was not sufficient to obtain maximal translocation into the SecYEG proteoliposomes (lane 4).

The stimulating effect by the *E. coli* SRP/SR system on the translocation of pSfmC and pTorT into SecYEG proteoliposomes was obtained upon the simultaneous addition of Ffh and FtsY but without adding extra 4.5S RNA. Figure 2B breaks down the individual contributions of all three components. Maximal translocation efficiency of pSfmC into SecYEG proteoliposomes was observed in the presence of Ffh, 4.5S RNA, FtsY, and SecA (lane 4). The omission of either Ffh (lane 8) or FtsY (lane 6) reduced the translocation efficiency to the level obtained by the mere addition of SecA (lane 2). Addition of 4.5S RNA, however, was not required (lane 14) due to some contamination of the purified Ffh with 4.5S RNA (unpublished observation) and of the commercial tRNA added to the PURE system, as previously demonstrated [21]. The results depicted in Figure 2A and B therefore indicate that also for secretory proteins harbouring hydrophobic signal sequences, SecA is necessary and sufficient for translocation. In contrast to classical secretory proteins such as OmpA, the presence of more hydrophobic signal sequences obviously requires the involvement of the bacterial SRP/SR system to achieve optimal translocation.

### Site-specific Photo Cross-linking Using the PURE System

We surmised that the beneficiary effect by SRP/SR on the translocation of SfmC might be mediated by the recruitment of Ffh to the hydrophobic signal sequence at the ribosome. In order to demonstrate this directly, we expanded the PURE system such as to allow the site-specific incorporation of the photo-activatable cross-linker *p*-benzoyl-phenylalanine (pBpa) into ribosome-associated nascent chains (RNCs) of SfmC via the suppression of amber stop codons engineered into the *sfmC* gene. To this end, a pBpa-accepting suppressor tRNA encoded by plasmid pEVOL-pBpF [22] was prepared by chloroform/phenol extraction and isopropanol fractionation of total tRNA from *E. coli* cells that had been transformed with pEVOL-pBpF. The same plasmid also encodes a pBpa-specific aminoacyl-tRNA synthetase, which was modified to contain a C-terminal His-tag to allow its purification and use in the PURE system. SfmC-RNCs could quantitatively be synthesized in an oligodeoxynucleotide-dependent manner [23] if the PURE system was deprived of all release factors.

### In the Absence of Membranes, the Signal Sequence of SfmC-RNCs is Found in Contact with Ffh

**Figure 3:**
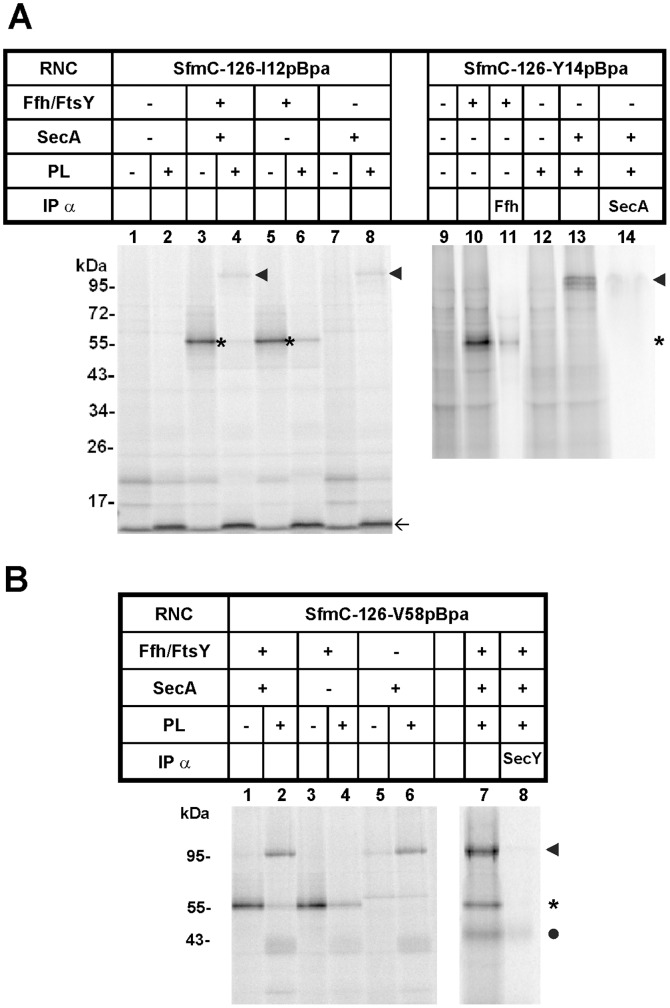
Co-translational cross-linking partners of SfmC. (**A**) RNCs of SfmC, 126 amino acids in length (*arrow*), carrying pBpa in their signal sequence either at position I12 or Y14 were synthesized by the PURE system (*SfmC-126-I12pBpa* and SfmC-126-Y14pBpa). *Ffh, FtsY, SecA*, and SecYEG proteoliposomes (*PL*) were present during synthesis as indicated. *Asterisks*, cross-links to Ffh (x Ffh); *arrow heads*, cross-links to SecA (x SecA). *IPα*, immunoprecipitation using the antibodies indicated. (**B**) as in (A), showing results obtained with SfmC-126 RNCs having pBpa incorporated at V58 located 35 residues downstream of the signal sequence cleavage site (*SfmC-126-V58pBpa*). *Dot*, cross-link to SecY (x SecY).

To identify binding partners of the SfmC signal sequence before and after membrane targeting, we synthesized SfmC-RNCs of 126 amino acid length that carried pBpa in the hydrophobic core of their signal peptide (Figure 3A, *arrow*). Upon supplementing the defined protein set of the PURE system with purified Ffh and FtsY, a prominent cross-linking product of the SfmC-RNCs appeared (Figure 3A, compare lanes 1 and 5, *asterisk*). Its size of about 60 kDa was indicative of an adduct between the SfmC-RNCs (about 13 kDa) and Ffh (48 kDa) and as such it was recognized by anti-Ffh antibodies (lane 11) and did not depend on the presence of FtsY (not shown). The Ffh cross-link markedly decreased in intensity upon addition of SecYEG proteoliposomes, (compare lanes 5 to 6) and even disappeared almost completely when purified SecA was added together with Ffh, FtsY, and proteoliposomes (compare lanes 3 to 4). Under these conditions, a new high molecular mass adduct appeared (lane 4, *arrow head*) that was recognized by anti-SecA antibodies (lane 14). Contacts to SecA were, however, only obtained in the presence of SecYEG proteoliposomes (compare lanes 3 and 4, *arrow head*) implying that only membrane-associated SecA interacted with the signal sequence of the SfmC-RNCs in these conditions. The fact that the interaction with SecA was paralleled by a drastic decrease in Ffh contacts would be consistent with an SRP-dependent targeting to membrane-bound SecA. Nevertheless Ffh and FtsY were dispensable for the SecA contacts to form (lane 8, *arrow head*) in accordance with the finding depicted in figure 2 that SecA by itself is sufficient to promote translocation of SfmC into SecYEG proteoliposomes. Conversely, the virtually exclusive cross-linking of SfmC-RNCs to Ffh when SecYEG proteoliposomes were missing, points to a primary recognition by SRP of the SfmC signal sequence when emerging from the ribosome. Equivalent results, i.e. recognition of the nascent signal sequence by SRP and its apparent transfer to SecA in the presence of SecYEG proteoliposomes were obtained for longer and shorter nascent chains of SfmC (data not shown).

### Targeting of SfmC-RNCs to SecY

We then moved the cross-linker from the signal sequence downstream to position V58 in the early mature part of SfmC (Figure 3B). Much like with Bpa located in the signal sequence, the SfmC-RNCs-V58Bpa yielded a very prominent adduct of about 60 kDa (*asterisk*, lane 1) that was totally dependent on the addition of Ffh (cf. lane 5). Hence a stretch of SfmC extending from its signal sequence up to position V58 was exposed to Ffh after having emerged from the ribosome. The cross-linker in position V58 also yielded the prominent 100 kDa adduct if SecA and SecYEG proteoliposomes were provided (*arrow head*, compare lanes 2 and 4). A slight interaction of SecA with V58 in the early mature part of SfmC was obtained even in the absence of SecYEG proteoliposomes, provided that the competing Ffh was missing (compare lanes 1 and 5). This finding is in line with the idea that SecA might interact with nascent chains due to its association with ribosomes (see Discussion). The shift of the cross-linking partners from Ffh to SecA observed upon addition of SecYEG proteoliposomes (compare lanes 1 and 2) would be expected if Ffh/FtsY was to target SfmC-RNCs to the SecYEG translocon. In fact, SfmC-RNCs with pBpa at position V58 also efficiently cross-linked to a resident protein of the SecYEG proteoliposomes (*dot*), which by immunoprecipitation was identified as SecY (lane 8).

## Discussion

Signal sequences, whose hydrophobicity exceeds a certain threshold level, have been proposed to direct secretory proteins to the SRP-dependent co-translational export pathway of *E. coli* [14], [15], [16]. This was deduced first from the diminished periplasmic export of these secretory proteins observed specifically when the Ffh protein was inactivated [14]. Secondly it was shown that in contrast to classical signal sequences, highly hydrophobic signal peptides can mediate export of a non-secretory protein, suggesting that by fusing a cytosolic protein with a hydrophobic signal sequence it can be withdrawn from folding in the cytosol through rerouting it to the SRP-dependent co-translational export pathway [15].

By use of an experimental system that allows studying the individual contributions of Ffh, FtsY and SecA to translocation across the SecYEG translocon, we were now able to establish the dominant role of SecA in the export of this kind of secretory proteins. Thus we could demonstrate that SecA is even sufficient for their translocation, while SRP and SR turned out to render transport more efficient. The latter finding would be consistent with the notion that because of inherently rapid folding kinetics, some secretory proteins require a hydrophobic signal sequence in order to co-translationally enter their export route and thereby escape premature folding [14].

Channeling secretory proteins into the SRP/SR-dependent co-translational pathway invokes their recognition early during translation at the ribosome. We therefore analyzed RNCs carrying a protein with a hydrophobic signal sequence (SfmC) and could show a definite interaction of its signal sequence with the Ffh protein, which is in clear contrast to our previous data obtained with the less hydrophobic signal sequence of OmpA [23], [24]. By virtue of its specific binding to the ribosomal protein L23 located at the orifice of the ribosomal exit tunnel, SecA was recently proposed to interact with nascent chains of classical secretory proteins, such as maltose-binding protein and β-lactamase, in order to increase the efficiency of their posttranslational targeting [25]. In fact, binding of SecA to a classical signal sequence had been shown to occur within a certain size frame of short nascent OmpA chains even in the presence of Ffh [23]. Under similar experimental conditions, the SfmC-RNCs analysed here did not interact with SecA whenever Ffh was present. This would be consistent with the predominant recognition of the more hydrophobic signal peptide of SfmC by SRP as suggested by previous *in vivo* analyses [14]. Only in the experimentally established absence of Ffh, a contact between SecA and SfmC-RNCs was detected and involved predominantly membrane-bound SecA. Thus cytosolically located SecA might play only a minor role as a targeting factor for proteins such as SfmC, whereas SfmC-RNCs can likely be targeted to the membrane through SecYEG-associated SecA, which *in vivo* is present at about 10fold higher concentrations than Ffh [1]. Collectively, our *in vitro* analysis employing highly defined experimental conditions demonstrate that the SRP dependence of secretory proteins with highly hydrophobic signal sequences is a result of a co-translational recognition of the hydrophobic signal sequence but that it is no prerequisite for the translocation of those secretory proteins, which can be executed solely by SecA.

## Materials and Methods

### Strains and Plasmids

*E. coli* strains DH5α [23], BL21 (DE3) (Novagen), M15 (Qiagen) were used for the preparation of plasmids and overexpression of proteins, and strain MC4100 [26] for the preparation of ribosomes. Plasmids pET19b-SecA [13], pTrc99a-Ffh, pTrc99a-FtsY, and pTrc99a-SecY_His_EG all described in ref. [27] were used to purify the corresponding proteins. Plasmid pEVOL-pBpF [22] (Addgene plasmid 31190) was used for purification of pBpa-tRNA-synthetase and pBpa-specific amber suppressor tRNA. 4.5S RNA was obtained by *in vitro* transcription of plasmid pT7/T3α19 [28].

The precursors of OmpA, SfmC and TorT were synthesized *in vitro* from plasmids pKSM717-OmpA [23], pET-SfmC and pET-TorT, respectively. To construct plasmids pET-SfmC and pET-TorT, the *sfmC* and *torT* genes were amplified from plasmids pCA24N-SfmC and pCA24N-TorT [29] using the primers SfmCfor, SfmCrev, TorTfor, TorTrev (Table 2). The PCR products were digested with NdeI and HindIII and integrated into the NdeI/HindIII- digested vector pET22b(+) to give plasmids pET-SfmC and pET-TorT.

**Table 2:** Primers used.

SfmCfor	GTGCATATGATGACTAAAATAAAGTTA
SfmCrev	AGTAAGCTTTTAGTTTAAGTTCACTTC
TorTfor	GATCATATGCGCGTACTGCTATTTTTA
TorTrev	ATCAAGCTTTTATTTCTTAGCCGCTGA
SfmCI12for	AAGTTATTGATGCTCATTTAGTTTTATTTAATCATTTCG
SfmCI12rev	CGAAATGATTAAATAAAACTAAATGAGCATCAATAACTT
SfmCY14for	TTGATGCTCATTATATTTTAGTTAATCATTTCGGCCAGC
SfmCY14rev	GCTGGCCGAAATGATTAACTAAAATATAATGAGCATCAA
pBpaRSHisfor	CCAATTAGAAAGAGATTAAAAGTCGACCATCATCATCAT
pBpaRSHisrev	ATGATGATGATGGTCGACTTTTAATCTCTTTCTAATTGG

TAG amber stop codons were introduced by mutagenizing PCR into the *sfmC* gene of pET-SfmC at positions encoding I12 and Y14 within the signal sequence (Phusion High-Fidelity DNA Polymerase, NEB) using the primers SfmCI12for, SfmCI12rev, SfmCY14for, SfmCY14rev (Table 2). Similarly, the pBpa-tRNA-synthetase gene of plasmid pEVOL-pBpF was modified to encode a C-terminal His-tag using the primers pBpaRSHisfor and pBpaRSHisrev (Table 2) generating plasmid pEVOL-pBpF-His.

### Purification of Proteins

Purification of SecA, Ffh, FtsY, pBpa-tRNA-synthetase and His-tagged components of the PURE system [17] were performed as described previously [4] with some modifications. Proteins were affinity-purified via their His-tags using TALON Metal Affinity Resins (Clontech) and 200 mM imidazole for elution. Ffh was stored at −20°C in HT buffer (50 mM HEPES-KOH, pH 7.6, 100 mM potassium acetate, 10 mM magnesium acetate, 1 mM dithiothreitol) supplemented with 50% glycerol. Other purified proteins were stored at −70°C in HT buffer supplemented with 30% glycerol. The SecYEG complex was purified as described [4].

### Isolation of Ribosomes

High salt-washed ribosomes were prepared as described [23] except that prior to sucrose gradient centrifugation, ribosomes were spun through a 1.44 M sucrose cushion.

### Reconstitution of Proteoliposomes

Preparation of SecYEG proteoliposomes was performed as described [13] except that 10 μg of purified SecYEG were reconstituted with 400 μg of *E. coli* phospholipids (Avanti Polar Lipids, Alabaster, AL).

### Protein Synthesis *in Vitro*

*In vitro* transcription and translation assays were performed in the PURE system as described previously [18] except for some modifications. The total volume of each reaction was changed to 25 μl and the concentration of magnesium acetate to 13 mM. Non-radioactive methionine and cysteine were provided at 4 mM each. The purified components of the PURE system were used at the following final concentrations: 68.79 μg/mL AlaRS (alanyl-tRNA synthetase), 2 μg/mL ArgRS, 22 μg/mL AsnRS, 7.97 μg/mL AspRS, 1.23 μg/mL CysRS, 3.79 μg/mL GlnRS, 12.63 μg/mL GluRS, 9.6 μg/mL GlyRS, 0.8 μg/mL HisRS, 39.53 μg/mL IleRS, 4.02 μg/mL LeuRS, 6.4 μg/mL LysRS, 2.08 μg/mL MetRS, 16.52 μg/mL PheRS, 10.24 μg/mL ProRS, 1.87 μg/mL SerRS, 6.29 μg/mL ThrRS, 1.05 μg/mL TrpRS, 0.61 μg/mL TyrRS, 1.81 μg/mL ValRS, 20 μg/mL MTF (methionyl-tRNA transformylase), 10 μg/mL IF1 (initiation factor 1), 40 μg/mL IF2, 10 μg/mL IF3, 50 μg/mL EF-G (elongation factor G), 100 μg/mL EF-Tu, 50 μg/mL EF-Ts, 10 μg/mL RF1 (release factor 1), 10 μg/mL RF2, 10 μg/mL RF3, 10 μg/mL RRF (ribosome recycling factor). RF1, RF2, RF3 and RRF were omitted when nascent chains were produced.

### Synthesis of RNCs

For the synthesis of ribosome-associated nascent SfmC chains of 126 amino acid length, 4 μg of the oligodeoxynucleotide GATAAAATCGCCAGTTGCAAAAC, 0.3 μg anti-10Sa RNA oligodeoxynucleotide TTAAGCTGCTAAAGCGTAGTTTTGGTCGTTTGCGACTA and 3 units RNaseH were additionally supplied in each 25 μl reaction according to [24].

### Photo Cross-linking

For the site-specific incorporation of pBpa into amber stop codon variants of SfmC, 80 μM pBpa, 20 μg/mL pBpa-tRNA synthetase, and 40 μg/mL pBpa-tRNA^sup^ prepared as described [30] were additionally supplied in each 25 μl reaction. After incubation at 37°C for 1 hour, the samples were exposed to UV irradiation at 365 nm for 20 min on ice.

### Miscellaneous

Protein translocation was analyzed by proteinase K resistance [31]. Immunoprecipitations were performed as described [13] except that after denaturation with SDS, samples were freed of precipitated material by centrifugation in a tabletop microfuge.

## References

[1] Kudva R, Denks K, Kuhn P, Vogt A, Müller M, et al (2013) Protein translocation across the inner membrane of Gram-negative bacteria: the Sec and Tat dependent protein transport pathways. Res Microbiol 164: 505–534. 23567322 10.1016/j.resmic.2013.03.016

[2] Zhang X, Rashid R, Wang K, Shan SO (2010) Sequential checkpoints govern substrate selection during cotranslational protein targeting. Science 328: 757–760. 20448185 10.1126/science.1186743 PMC3760334

[3] Ataide SF, Schmitz N, Shen K, Ke A, Shan SO, et al (2011) The crystal structure of the signal recognition particle in complex with its receptor. Science 331: 881–886. 21330537 10.1126/science.1196473 PMC3758919

[4] Braig D, Mircheva M, Sachelaru I, van der Sluis EO, Sturm L, et al (2011) Signal sequence-independent SRP-SR complex formation at the membrane suggests an alternative targeting pathway within the SRP cycle. Mol Biol Cell 22: 2309–2323. 21551068 10.1091/mbc.E11-02-0152 PMC3128533

[5] Bibi E (2012) Is there a twist in the *Escherichia coli* signal recognition particle pathway? Trends Biochem Sci 37: 1–6. 22088262 10.1016/j.tibs.2011.09.004

[6] Qi HY, Bernstein HD (1999) SecA is required for the insertion of inner membrane proteins targeted by the *Escherichia coli* signal recognition particle. J Biol Chem 274: 8993–8997. 10085146 10.1074/jbc.274.13.8993

[7] Tian H, Boyd D, Beckwith J (2000) A mutant hunt for defects in membrane protein assembly yields mutations affecting the bacterial signal recognition particle and Sec machinery. Proc Natl Acad Sci U S A 97: 4730–4735. 10781078 10.1073/pnas.090087297 PMC18301

[8] Neumann-Haefelin C, Schäfer U, Müller M, Koch HG (2000) SRP-dependent co-translational targeting and SecA-dependent translocation analyzed as individual steps in the export of a bacterial protein. EMBO J 19: 6419–6426. 11101515 10.1093/emboj/19.23.6419 PMC305875

[9] van der Laan M, Nouwen N, Driessen AJ (2004) SecYEG proteoliposomes catalyze the Deltapsi-dependent membrane insertion of FtsQ. J Biol Chem 279: 1659–1664. 14578344 10.1074/jbc.M306527200

[10] Deitermann S, Sprie GS, Koch HG (2005) A dual function for SecA in the assembly of single spanning membrane proteins in *Escherichia coli* . J Biol Chem 280: 39077–39085. 16186099 10.1074/jbc.M509647200

[11] Scotti PA, Valent QA, Manting EH, Urbanus ML, Driessen AJ, et al (1999) SecA is not required for signal recognition particle-mediated targeting and initial membrane insertion of a nascent inner membrane protein. J Biol Chem 274: 29883–29888. 10514469 10.1074/jbc.274.42.29883

[12] Antonoaea R, Fürst M, Nishiyama K, Müller M (2008) The periplasmic chaperone PpiD interacts with secretory proteins exiting from the SecYEG translocon. Biochemistry 47: 5649–5656. 18439025 10.1021/bi800233w

[13] Welte T, Kudva R, Kuhn P, Sturm L, Braig D, et al (2012) Promiscuous targeting of polytopic membrane proteins to SecYEG or YidC by the *Escherichia coli* signal recognition particle. Mol Biol Cell 23: 464–479. 22160593 10.1091/mbc.E11-07-0590 PMC3268725

[14] Huber D, Boyd D, Xia Y, Olma MH, Gerstein M, et al (2005) Use of thioredoxin as a reporter to identify a subset of *Escherichia coli* signal sequences that promote signal recognition particle-dependent translocation. J Bacteriol 187: 2983–2891. 15838024 10.1128/JB.187.9.2983-2991.2005 PMC1082830

[15] Schierle CF, Berkmen M, Huber D, Kumamoto C, Boyd D, et al (2003) The DsbA signal sequence directs efficient, cotranslational export of passenger proteins to the *Escherichia coli* periplasm via the signal recognition particle pathway. J Bacteriol 185: 5706–5713. 13129941 10.1128/JB.185.19.5706-5713.2003 PMC193964

[16] Shimohata N, Akiyama Y, Ito K (2005) Peculiar properties of DsbA in its export across the *Escherichia coli* cytoplasmic membrane. J Bacteriol 187: 3997–4004. 15937162 10.1128/JB.187.12.3997-4004.2005 PMC1151732

[17] Shimizu Y, Inoue A, Tomari Y, Suzuki T, Yokogawa T, et al (2001) Cell-free translation reconstituted with purified components. Nat Biotechnol 19: 751–755. 11479568 10.1038/90802

[18] Holzapfel E, Moser M, Schiltz E, Ueda T, Betton JM, et al (2009) Twin-arginine-dependent translocation of SufI in the absence of cytosolic helper proteins. Biochemistry 48: 5096–5105. 19432418 10.1021/bi900520d

[19] Kuruma Y, Nishiyama KI, Shimizu Y, Müller M, Ueda T (2005) Development of a Minimal Cell-Free Translation System for the Synthesis of Presecretory and Integral Membrane Proteins. Biotechnol Prog 21: 1243–1251. 16080708 10.1021/bp049553u

[20] Nishiyama K, Maeda M, Abe M, Kanamori T, Shimamoto K, et al (2010) A novel complete reconstitution system for membrane integration of the simplest membrane protein. Biochem Biophys Res Commun 394: 733–736. 20230783 10.1016/j.bbrc.2010.03.061

[21] Koch HG, Hengelage T, Neumann-Haefelin C, MacFarlane J, Hoffschulte HK, et al (1999) In vitro studies with purified components reveal signal recognition particle (SRP) and SecA/SecB as constituents of two independent protein-targeting pathways of *Escherichia coli* . Mol Biol Cell 10: 2163–2173. 10397756 10.1091/mbc.10.7.2163 PMC25430

[22] Young TS, Ahmad I, Yin JA, Schultz PG (2010) An enhanced system for unnatural amino acid mutagenesis in *E. coli* . J Mol Biol 395: 361–374. 19852970 10.1016/j.jmb.2009.10.030

[23] Eisner G, Koch HG, Beck K, Brunner J, Müller M (2003) Ligand crowding at a nascent signal sequence. J Cell Biol 163: 35–44. 14530384 10.1083/jcb.200306069 PMC2173441

[24] Beck K, Wu LF, Brunner J, Müller M (2000) Discrimination between SRP- and SecA/SecB-dependent substrates involves selective recognition of nascent chains by SRP and trigger factor. EMBO J 19: 134–143. 10619852 10.1093/emboj/19.1.134 PMC1171785

[25] Huber D, Rajagopalan N, Preissler S, Rocco MA, Merz F, et al (2011) SecA interacts with ribosomes in order to facilitate posttranslational translocation in bacteria. Mol Cell 41: 343–353. 21292166 10.1016/j.molcel.2010.12.028

[26] Casadaban MJ, Cohen SN (1979) Lactose genes fused to exogenous promoters in one step using a Mu-lac bacteriophage: in vivo probe for transcriptional control sequences. Proc Natl Acad Sci U S A 76: 4530–4533. 159458 10.1073/pnas.76.9.4530 PMC411611

[27] Kuhn P, Weiche B, Sturm L, Sommer E, Drepper F, et al (2011) The bacterial SRP receptor, SecA and the ribosome use overlapping binding sites on the SecY translocon. Traffic 12: 563–578. 21255212 10.1111/j.1600-0854.2011.01167.x

[28] Wood H, Luirink J, Tollervey D (1992) Evolutionary conserved nucleotides within the *E.coli* 4.5S RNA are required for association with P48 in vitro and for optimal function in vivo. Nucleic Acids Res 20: 5919–5925. 1281314 10.1093/nar/20.22.5919 PMC334455

[29] Kitagawa M, Ara T, Arifuzzaman M, Ioka-Nakamichi T, Inamoto E, et al (2005) Complete set of ORF clones of *Escherichia coli* ASKA library (a complete set of *E. coli* K-12 ORF archive): unique resources for biological research. DNA Res 12: 291–299. 16769691 10.1093/dnares/dsi012

[30] Deutscher MP, Hilderman RH (1974) Isolation and partial characterization of *Escherichia coli* mutants with low levels of transfer ribonucleic acid nucleotidyltransferase. J Bacteriol 118: 621–627. 4597452 10.1128/jb.118.2.621-627.1974 PMC246796

[31] Moser M, Panahandeh S, Holzapfel E, Müller M (2007) In vitro analysis of the bacterial twin-arginine-dependent protein export. Methods Mol Biol 390: 63–80. 17951681 10.1007/978-1-59745-466-7_5

